# *In Silico* and *In Vitro* Investigations of the Mutability of Disease-Causing Missense Mutation Sites in Spermine Synthase

**DOI:** 10.1371/journal.pone.0020373

**Published:** 2011-05-27

**Authors:** Zhe Zhang, Joy Norris, Charles Schwartz, Emil Alexov

**Affiliations:** 1 Computational Biophysics and Bioinformatics, Department of Physics, Clemson University, Clemson, South Carolina, United States of America; 2 J.C. Self Research Institute of Human Genetics, Greenwood Genetic Center, Greenwood, South Carolina, United States of America; 3 Department of Genetics and Biochemistry, Clemson University, Clemson, South Carolina, United States of America; University of South Florida, United States of America

## Abstract

**Background:**

Spermine synthase (SMS) is a key enzyme controlling the concentration of spermidine and spermine in the cell. The importance of SMS is manifested by the fact that single missense mutations were found to cause Snyder-Robinson Syndrome (SRS). At the same time, currently there are no non-synonymous single nucleoside polymorphisms, nsSNPs (harmless mutations), found in SMS, which may imply that the SMS does not tolerate amino acid substitutions, i.e. is not mutable.

**Methodology/Principal Findings:**

To investigate the mutability of the SMS, we carried out *in silico* analysis and *in vitro* experiments of the effects of amino acid substitutions at the missense mutation sites (G56, V132 and I150) that have been shown to cause SRS. Our investigation showed that the mutation sites have different degree of mutability depending on their structural micro-environment and involvement in the function and structural integrity of the SMS. It was found that the I150 site does not tolerate any mutation, while V132, despite its key position at the interface of SMS dimer, is quite mutable. The G56 site is in the middle of the spectra, but still quite sensitive to charge residue replacement.

**Conclusions/Significance:**

The performed analysis showed that mutability depends on the detail of the structural and functional factors and cannot be predicted based on conservation of wild type properties alone. Also, harmless nsSNPs can be expected to occur even at sites at which missense mutations were found to cause diseases.

## Introduction

Spermine Synthase (SMS) is an enzyme which converts spermidine (SPD) into spermine (SPM), both of which are polyamines and play an essential role in normal mammalian cell growth and development [1], [2], [3], [4]. Their synthesis requires the presence of variety of compounds including decarboxylated S-adenosylmethionine (DCAdoMet) and decarboxylased ornithine (ODC). Thus a gene deletion of S-adenosylmethionine decarboxylase (AdoMetDC), which generates DCAdoMet, resulted in fatal embryonic development indicating that polyamines are required for cell proliferation in the embryo [5]. Similarly, a deletion of the ornithine decarboxylase gene illustrated that ornithine decarboxylase is essential for cell survival during early murine development [6]. Another series of experiments indicated that Gy male mice are of smaller size and have higher mortality by weaning age than normal male littermates [7]. All these examples confirm the importance of polyamines and a disruption of the enzymes in polyamine biosynthetic pathways results in abnormal cell development. Because of their critical role in the cell, polyamine biosynthetic enzymes are frequently drug targets [8], [9].

The importance of SMS for normal cell functioning is illustrated by the fact that the malfunctioning of SMS is associated with the Snyder-Robinson Syndrome (SRS, OMIM 309583), which is an X-linked recessive disease consisting of mild-to-moderate mental retardation, osteoporosis, facial asymmetry, thin habitus, hypotonia, and a nonspecific movement disorder [10], [11], [12]. Further, missense mutations in SMS were shown to affect the brain morphology as indicated by volumetric neuroimaging analyses [13]. In addition, deficiency of SMS in mice was demonstrated to cause deafness [14]. Currently, there are no reported non synonymous single nucleoside polymorphisms (nsSNPs) in SMS. These are presumably harmless mutations found in the general population. Could this be a consequence of a potential resistivity of SMS to amino acid substitutions? This is a question that the present study attempts to address.

Recently, the 3D structure of human SMS has been determined and it was shown that human SMS has two subunits forming a homo-dimer [15]. Each subunit has two functional domains: C-domain and N-domain. Structural and biochemical analyses showed that the active site is located within the C-domain, while N-domain is critical for dimerization, which in turn is required for normal function of the SMS [15]. We took advantage of the available 3D structure and experimental data and carried out an *in silico* analysis of the effects of the missense mutations, p.G56S (c.267G>A) [12], p.V132G (c.496T>G) [10] and p.I150T (c.550T>C), which are known to cause SRS [16]. Our work showed that the mutations affect dimer and monomer stability and perturb the hydrogen bond network of the functionally important amino acids. However, no attempt has been made to assess the mutability of these sites and to address the possibility that other amino acid substitutions, which are different from those known to cause SRS, could potentially cause SRS or, more generally, be harmless.

**Figure 1:**
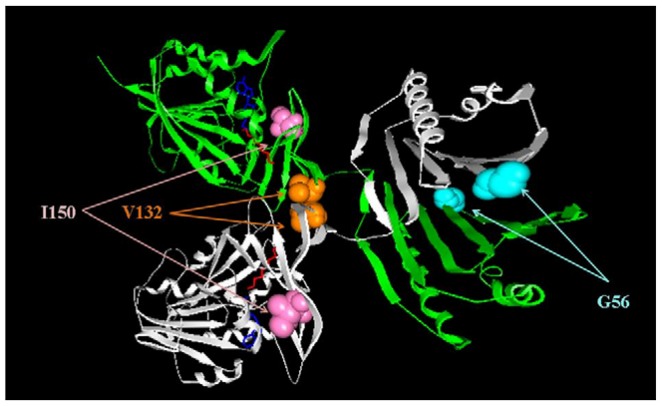
3D structure of SMS with the three mutation sites shown as colored balls.

nsSNPs and missense mutations can affect wild type protein function by a variety of mechanisms [17], [18], [19], however, the most common effect is destabilization of the native structure [20], [21], [22], [23], [24], [25], altering macromolecular interactions [26] or affecting wild type hydrogen bond patterns [16], [27], [28]. This study focuses on predicting the effects of amino acid substitutions on these three important native characteristics of SMS. To make the task computationally tractable, the mutational analysis is restricted to the sites (G56, V132, and I150) which are clinically known to harbor missense mutations causing SRS (Figure 1). We mutate *in silico* the wild type residue at these positions to each other amino acid and predict the effect on stability of the SMS monomer, affinity of SMS dimer, the ionization states, and the hydrogen bond network within SMS.

## Methods

### Protein structures

The X-ray crystal structure of the wild type human SMS protein, in complex with spermidine and 5-methylthioadenosine (MTA) (PDB ID 3C6K), was downloaded from the Protein Data Bank (PDB) website [29]. The asymmetrical complex has four subunits, while the biological unit is a dimer. The dimer, which is formed of A and B subunits, was found to have significant van der Waals clashes and was omitted from the analysis. Missing atoms and residues were rebuilt with profix program from Jackal package (http://wiki.c2b2.columbia.edu/honiglab_public/index.php/Software:Jackal_General_Description). The WT residues at positions 56, 132, and 150 (Figure 1) were mutated in *silico* using the default parameters (without minimization) of the program SCAP [30]. Note that in this paper we use the original residue numbers reported in the literature and in 3C6M PDB file, while the corresponding residue numbers in 3C6K PDB file are shifted by 2 and are reported in parentheses (the corresponding mutation sites are G58, V134 and I152 respectively).

### Energy calculations

Due to SMS being such a large protein, full-scale energy calculations are computationally demanding, a single energy minimization run taking more than two weeks to complete on a typical Linux cluster. In addition, our experience indicates that the inclusion of the structural domains that are far from the mutation site degrades the accuracy of the calculations as indicated through the RMSD of the obtained energies with different force fields (Zhang et al., unpublished results). Because of this potential for degradation, the SMS protein was split into two domains: (a) The N-domain including residues 2–109 (4–111 in 3C6K) and (b) C-domain containing residues 118–364 (120–366 in 3C6K). The substrates (SPD and MTA) were removed from the C-domain file to simplify the runs, since the setup required manual intervention for each of the mutants. However, we performed a test run for the WT and the I150T mutant, and the inclusion of the substrates had a minimal effect on the outcome of the energy results.

#### (a) binding free energy calculations

The energies of the structures of the corresponding domains were minimized using the “minimize.x” module from TINKER package [31] applying the Limited Memory BFGS Quasi-Newton Optimization algorithm and convergence criterion of 0.01. The solvent was modeled with the Still Generalized Born model [32]. The protein internal dielectric constant was 1.0. For each dimer, three separate runs were performed using three different force fields, Amber98 [33], Charmm27 [34] and Oplsaa [35]. This was done to deliver results less sensitive to a particular force field and to provide a criterion for selecting the optimal computational protocol. After the energy was successfully minimized, the structures of the monomers (C and D) were taken from the energy minimized dimer. After applying a protocol, which was termed the “rigid body” protocol, the minimization was found to be much less sensitive to the choice of a particular force field as compared with a protocol that minimizes monomers independently from the dimer.

The structures of the energy minimized dimer, the C and D monomers, were then subjected to the “analyze.x” module of TINKER to calculate the total potential energy of the system. Thus, the binding free energy of both WT and each mutant was calculated by the following formula:
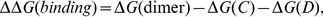
(1)where 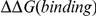 is the binding free energy; ΔG(dimer) is the total potential energy of the dimer; ΔG(C) and ΔG(D) are the potential energies of monomer “C” and “D”, respectively.

The effect of the mutation on the affinity (binding) was calculated by [16], [26]:
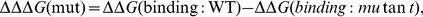
(2)where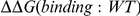 is the binding free energy of the WT dimer and 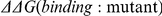 is the binding energy of the corresponding mutant. We assume that the entropy change associated with the dimer formation is practically the same for the WT and mutant. Therefore, the entropy change cancels out in both of the above formulas (1) and (2).

#### (b) folding free energy calculations

The stability (folding free energy) of individual monomers was calculated using following protocol. Each truncated monomer (see above the boundaries of domains), C or D, was energy minimized using the same protocol as was applied to the dimer minimization. After successful minimization, a structural segment of a length of seven (different lengths were tested as well) amino acids, with the mutation site at the center, was extracted from the minimized structure. Then the minimized structure of the corresponding monomer and the seven residue segment were subjected to the “analyze.x” module of TINKER to obtain the total potential energy.

The folding free energy calculations follow the approach described in our original paper [16]. The potential energy of each truncated monomer *G(folded)* is the potential energy obtained with TINKER using the corresponding energy minimized structure. Having in mind that our investigation focuses on single mutations, the unfolded state was considered to be quite similar for the wild type and the mutant SMS as described in our previous work [16]. Thus, amino acids away from the site of mutation were assumed to adopt the same unfolded state in both the wild type and the mutant. The free energy associated with this part of unfolded protein is termed G_0_ (unfolded). The difference of the unfolded states between the wild type and mutant SMS was modeled by taking seven residue segment (different lengths up to nine residues were tested, but no significant effect was found) centered at the site of mutation. The corresponding energy is termed G_7_ (unfolded) and was calculated with TINKER using the energy minimized structure of seven residue segment. Then the folding free energy is: 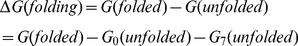
(3)where *G(folded)* is the total potential energy of the folded state and *G(unfolded)* is the energy of the unfolded state.

The effect of a mutation is calculated as:
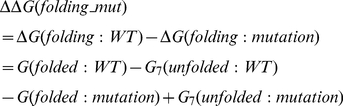
(4)


Since G_0_ (unfolded) is mutation independent, it cancels out in eq. (4). Such an approach avoids the problems with modeling unfolded states since it focuses on the energy difference between WT and mutant structures. Here we assumed that the unfolded state of the WT and mutants are almost the same except for the seven residue segments centered at the missense mutation site and residues of the mutation site do not interact with the further amino acids. The applicability of such an approach with respect to the experimental results of the effect of mutation on the melting temperature was reported in Ref. [36].

### pKa calculations

A point mutation can dramatically change the hydrogen bond network and even cause a change of the ionization states of neighboring amino acids. To investigate such possibilities, the Multi Conformation Continuum Electrostatics (MCCE), version 2.4 (http://134.74.90.158/) [37], [38], [39] was used to perform pKa calculations on the WT monomers and the corresponding mutants. The MCCE calculates the ionization states of titratable groups and optimizes the hydrogen bond network. Default parameters of MCCE were used. The substrates, SPD, and MTA were included in the calculations for C-terminal domain. The reason for these inclusions is the long range of electrostatic interactions, which are the main source of the pKa changes. The corresponding pKa shifts were calculated as:
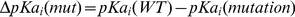
(5)where *pKa_i_(WT)* is the *pKa* of the *ith* amino acid calculated with the WT structure while *pKa_i_(mutation)* is the *pKa* of the same amino acid calculated using the mutant structure.

### Z-Score

In statistics, a standard score (Z-Score) indicates how many standard deviations an observation or datum is above or below the mean. The quantity Z represents the distance between the raw score and the population mean in units of the standard deviation. Z-score is negative when the raw score is below the mean and positive when above (see Wikipedia: http://en.wikipedia.org/wiki/Standard_score). In our case, the distribution is constructed from either the ΔΔΔG(mut) (the change of the binding free energy, eq. 2) or ΔΔG(folding_mut) (the change of the folding free energy, eq. 4).
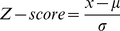
(6)where:

*x* is a raw score to be standardized;

*µ* is the mean of the population;

*σ* is the standard deviation of the population

In this work, we use Z-Score to reflect the mutability of the mutation sites on stability of the monomers and affinity of the dimer. A large Z-score indicates a mutation that causes an effect significantly different from the average effect at the site.

### Classification nomenclature

The main goal of this work is to probe (*in silico*) the mutability of sites in the SMS harboring missense mutations causing SRS. It is accomplished by performing energy and pKa calculations. However, currently there is no metric which suggests how big the energy or pKa change should be in order to consider a given mutation to be disease causing or not. Perhaps such a threshold will be case dependent. However, in order to provide a better quantitative description of our results we introduce two terms:

- “tolerance” – if the mean of the distribution of the energy change (either binding or folding free energy change) upon amino acid substitutions at a given site is larger than the half of the standard deviation (HSTD) for the site (Supporting information, Tables S1 and S2), then the site is classified as a “non-tolerable” site. Otherwise it is termed “tolerable”. In the case of pKa calculations, the cut-off is taken to be 2pK units cumulated over all titratable groups. This threshold is selected empirically.
- “specificity” – a site is termed “specific” if more than 20% of amino acid substitutions are predicted to cause different effects from favorable to unfavorable energy change with a magnitude larger than the half of the standard deviation (HSTD) associated with the site (Tables S1 and S2 in supplementary material). If the effects follow the same trend, then the site is termed “non-specific”. In terms of pKa calculations, a site is termed “specific” if the pKa shifts among different types of substitutions differ by more than 2 pK units (empirically selected threshold).

### Experiments in vitro

#### (a) RNA

RNA was prepared from a control cell line and from patient lymphoblast cell lines [11] using GenElute Mammalian Total RNA Miniprep kit (Sigma catalogue number RTN-70).

#### (b) Plasmids Construction

cDNA was prepared with Superscript First strand Synthesis Kit for RT-PCR (Invitrogen catalogue number 11904-018) from 2u g of RNA prepared from lymphoblast cell lines. The gene specific oligo SMS-RT (5′ GAA GGC TAT TTG CAG CAC ATG TGA 3′), was used to generate the first strand cDNA for the control and G56S, V132G, and I150T mutations. A PCR reaction with the oligos SMS-F (5′ CAC CAT GGC AGC AGC ACG GCA CAG CAC G 3′) and SMS-R (5′ GGG TTT AGC TTT CTT CCA AAC AGT 3′) and PFU Turbo (Stratagene catalogue number 600250) was employed to generate the insert. The fragment was run on a 1% TAE agarose gel and purified with a Gel Extraction Kit (Qiagen catalogue number 28704). The purified product was cloned into pcDNA3.1D/V5-His-Topo vector using the pcDNA3.1 Directional Topo Expression Kit (Invitrogen catalogue number K4900-01). All plasmids generated from this kit have a V5 tag on the C-terminus. Plasmids were sequenced to confirm the insert with the vector specific primers PC (5′ GGG AGA CCC AAG CTG GCT AGT 3′) and BGH (5′ TAG AAG GCA CAG TCG AGG 3′) and insert specific primers SMS285For (5′GAG AAT TTA CCC ACA TGG ATT 3′), SMS330Rev (5′GTG GGC CAG TAT CTG TCG AT 3′), SMS660Rev (5′ GTC TCC ACC TCC CAG AAT GA 3′)and SMS960Rev (5′GGA GAC GTG GAG ATT GGA ACA 3′).

#### (c) Site-directed mutagenesis (Table S3)

Primers were designed to generate the various amino acid changes (sequences available upon request) in the control SMS construct using the QuikChange Primer Design Program online program provided by the Stratagene (http://www.stratagene.com/qcprimerdesig). Mutations were generated using the QuikChange II Site-directed Mutagenesis kit (Stratagene catalogue number 200523) and the specifically designed primers. All plasmids were sequenced to confirm the specific mutation mutations generated without additional changes.

#### (d) Cell culture

HEK cells were obtained from American Tissue Culture Collection. The cells were cultured in DMEM (Sigma catalogue number D5796) supplemented in 10%FBS (Atlanta Biologicals catalogue number S12450H), 1x Penicillin/Streptomycin (Sigma catalogue number P0781), 2 mM glutamine (Sigma catalogue number G7513) in a 5% CO_2_ humidified 37C incubator.

#### (e) Transfection

HEK cells were cultured on poly-l-lysine (Sigma catalogue number P4707) coated 24 well tissue culture dishes in growth media 18–24 hrs prior to transfection. A transfection complex containing one microgram of plasmid DNA and 2 ul of Lipofectamine 2000 (Invitrogen catalogue number 11668-027)/100 ul of DMEM was added to each well. After 4 hours, the transfection complexes were removed, the cells washed one time with PBS, and growth media was added to the cells.

#### (f) Native gel electrophoresis

Forty-eight hours after the transfection, the cells were washed twice with ice cold PBS (Sigma catalogue number D8537) and scrapped into 100 ul of ice cold native gel buffer(0.62 mM Tris HCL pH 6.8, 0.01% bromophenol blue, 10% glycerol). The samples were vortexed and sonicated briefly and kept on ice. Ten ul of each sample was separated on a 7.5% native polyacrylamide gel and transferred to nylon-supported nitrocellulose (Fisher catalogue number WP4HY330F5) using a Biorad Semi-dry Transfer apparatus at 24 volts for 1 hour. After western transfer, the nitrocellulose membrane was rinsed twice with TBST (25 mM Tris, 150 mM NaCl, 0.2% Tween 20, pH 8.0) for 5 minutes each.

#### (g) Western blot

Blocking buffer (5% nonfat dry milk in TBST) was added to the membrane and incubated at room temperature for 1 hour with shaking. The membrane was then incubated with Anti-V5 monoclonal antibody (Invitrogen catalogue number R960-25) diluted 1∶5000 in fresh blocking buffer at 4°C with shaking, overnight. The antibody solution was removed and the membrane was rinse three times for 20 minutes with TBST at room temperature with shaking. The membrane was incubated in anti-mouse IgG Horse Radish peroxidase conjugate (Pierce catalogue number 31432) diluted 1∶10,000 in 2% BSA (Sigma catalogue number A4503)/TBST for one hour with shaking at room temperature. The antibody solution was discarded and the membrane was rinsed three times with TBST for 20 minutes at room temperature with shaking. SuperSignal West Dura Extended Duration Substrate (Pierce catalogue number 34075) was added to the membrane for five minutes and the membrane exposed to autoradiography film (Midlands X-ray catalogue number agfaB). The film was processed on a Konica SRX 101A film developer.

## Results

### Effect of mutations on the stability of the monomers

**Figure 2:**
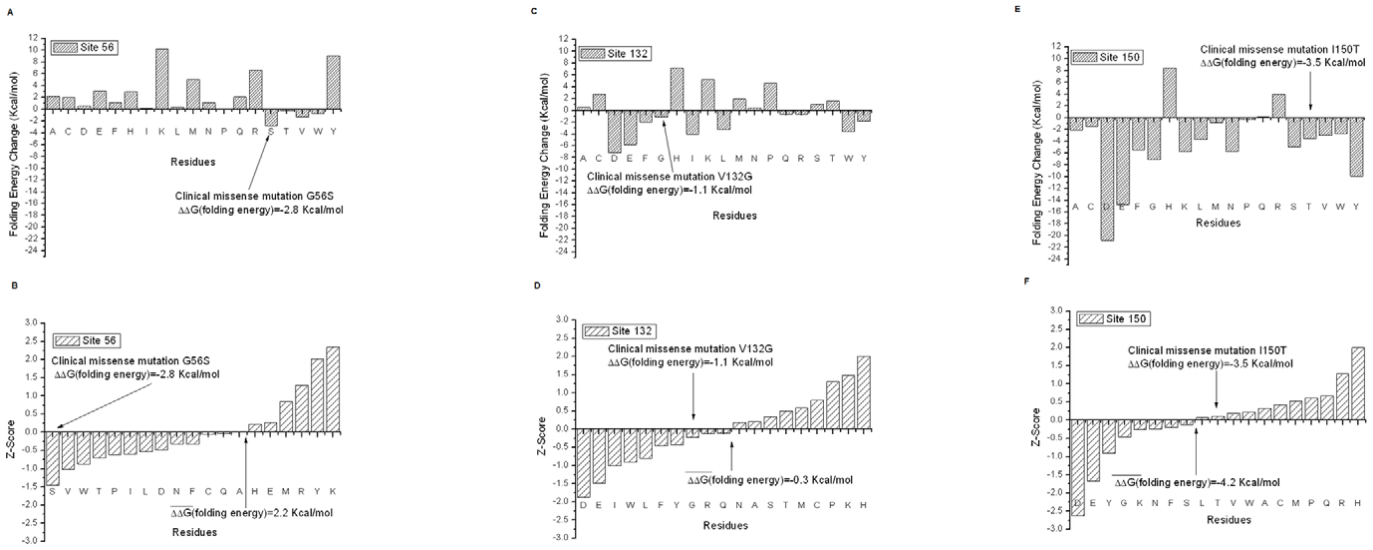
The results of structure-based energy calculations on the monomer stability with three different force field parameters (Charmm27, Amber98 and Oplsaa) and then results averaged for the clinically observed missense mutations G56S, V132G, and I150T, we used four force fields, which are Charmm27, Charmm19, Amber98 and Oplsaa, then results averaged). The left panels are the predicted energy changes of the folding free energy and the right panels are the Z-Scores of folding energy change. The 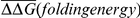 in the graph is the mean of folding energy change over the 19 mutations. The results are shown for G56 (a–b), V312 (c–d) and I150 (e–f) sites.

Figure 2 summarizes the results of structure-based energy calculations on the monomer stability with three different force field parameters (Charmm27, Amber98 and Oplsaa). The results of each force field were then averaged. The results were averaged over the C and D chains as well. All energies are in Kcal/mol. A negative energy change value indicates that the mutation decreases the stability of the monomer, while a positive value increases the stability. Below we analyze the outcome for each mutation site separately:

#### (a) G56 site

The G56 site is situated on a sharp loop connecting two beta strands and is almost totally exposed to the water phase in monomers. The fact that position 56 in the WT structure is occupied by a Gly residue is typically attributed to a necessity of the polypeptide chain to make a tight turn, while avoiding sterical constrains. However, the energy calculations (Figure 2A) indicate that many amino acid replacements have almost no effect on the stability or make the folding energy slightly bigger, including residues with large side chains, such as Trp and Ile. The structural reason for this is that there is enough room for these side chains to find relative favorable positions and to compensate for the stress induced by the sharp backbone turn. At the same time, there are several amino acids which are predicted to significantly increase the monomer's stability. The most prominent examples are Lys, Arg, and Tyr, which are expected to increase monomers stability by almost 10 Kcal/mol. This is due to the negative potential created by several acidic groups (Asp 77 (Asp 79 in 3C6K) and Asp 79 (Asp 81 in 3C6K)) and backbone oxygens of the residues in the loop where is the mutation site, which makes insertion of positively charged Lys and Arg energetically favorable. The Tyr mutation is favorable due to the relatively long side chain of Tyr that allows formation of H-bond with Gln 72 (Gln 74 in 3C6K). The most puzzling is the prediction that Met substitution should increase the monomers' stability, however, during the minimization process, its side chain manages to bend and point toward the protein's interior and thus to become energetically favorable.

Figure 2B shows the Z-scores, arranged in increasing order, of all amino acid substitutions at site 56. The mean of the distribution is a positive number, indicating that most of the substitutions are expected to increase the monomers' stability. The mean is 2.2 Kcal/mol, while the HSTD calculated for this site 1.9 Kcal/mol. Formally speaking the site should be classified as “non-tolerable”, but since the mean and the threshold are so close and because of many substitutions that cause almost no effect on stability (Figure 2A), it is classified as “tolerable”. The tolerability can be attributed to the mutations for which the amplitude of the folding energy change is relatively small (all cases with Z-score between 0.0 and -1.0). Because of this, many mutations will have no effect to the SMS folding energy, and perhaps, will not affect its function. There are only a few mutations that are predicted to destabilize the monomers, but the amplitude of the folding energy change is small, so the site is classified as “non-specific”. All together, site 56 is termed a “tolerable non-specific” site and is quite likely to be able to hold harmless variations, based on the folding energy alone.

#### (b) V132 site

In a monomeric state, the V132 site is exposed to the water and the side chain of the WT residue is parallel to the molecular surface, pointing toward a large cavity of the SMS protein. There are no hydrogen bond acceptors/donors in the close proximity of this site, but the site itself is situated within a strong negative electrostatic potential generated by several acidic groups. This negative potential, perhaps, is part of a large electrostatic funnel that guides the positively charged SPD inside the SMS to carry the reaction forward.

The results of the folding free energy calculations show mixed trends, some of the mutation destabilizing while other stabilizing monomeric structures (Figure 2C). Most of the mutations are predicted to cause very small perturbations of the folding energy, with several prominent exceptions. Negatively charged amino acids, Glu and Asp, are found to destabilize the monomers. This is due to the fact that the site is already within strong negative potential and introducing extra negative charge decreases the stability. Relatively short, positively charged amino acids, like His and Lys, take advantage of this and increase the monomers' stability.

The Z-score distribution (Figure 2D) reflects the above observations and has a mean of almost zero. Such a distribution will, according to our protocol, classify the V132 site as a “tolerable” site with respect to the folding free energy. At the same time, there are mutations predicted to cause opposite effects on the stability with a magnitude larger than the HSTD of 3.1 Kcal/mol. According to our nomenclature, such a site is a “tolerable specific” site. The negatively charged amino acids (Glu and Asp) were found at the left wing of the Z-score distribution, while the positively charged acids reside on the right side. This indicates that site V132 is quite sensitive to the polarity of the charge that is positioned there.

#### (c) I150 site

The side chain of Ile at position 150 is totally buried in the protein's interior and any mutation will result in a side chain buried as well. This site is well packed and does not allow for large structural reorganization upon amino acid substitution. It also experiences a strong negative potential coming from a large number of acidic residues in the neighborhood.

The results of the folding free energy calculations are shown in Figure 2E. It can be seen that almost any mutation is expected to greatly reduce the monomers' stability. As it should be expected, position 150 does not tolerate negatively charge amino acids – both Glu and Asp mutations are predicted to decrease the monomers' stability by more than 14 Kcal/mol. Polar and hydrophobic residue insertions have smaller effects (as compared with charge residues) on the stability. This is due to a combination of factors, as position 150 is buried, allowing for new hydrogen bond formations, but the space is very confined. Thus, some residues may manage to establish hydrogen bonds, as the clinically observed I150T, but they have to pay the desolvation cost, and thus the energy change is reduced. Only two substitutions are predicted to increase the monomers stability, His and Arg substitutions. This is due to their specific side chain geometry, which permits the formation of strong favorable interactions and in turn over compensates the desolvation cost.

The Z-score is shown in Figure 2F and the mean of the change of folding free energy distribution is a large negative number (HSTD is 2.6 Kcal/mol). Practically any mutation will significantly destabilize the monomeric structure. Such a site is referred to as a “non-tolerable non-specific” site with respect to the folding free energy. Similarly to the V132 site, the Asp and Glu residues are at the left wing of the Z-score distribution, indicating that site 150 does not support negative charge.

### Effect of mutations on the affinity of the dimer

Dimerization is absolutely required for the normal function of SMS as it was shown experimentally [15]. Therefore, any change of the dimer affinity caused by a mutation is expected to affect SMS function. Below we present the results of *in silico* modeling of the effects of mutations on the SMS dimer binding free energy.

#### (a) G56 site

Position G56 is at the periphery of the dimer binding interface but the side chain of any residue at this position will point toward the opposite partner. It should be clarified that while SMS is a homo-dimer, the interface is not symmetrical, i.e. site G56 is not symmetrical across the interface. Across the interface there are neither specific hydrogen donors or acceptors nor charged residues that could be involved in specific interactions with an amino acid at position 56.

**Figure 3:**
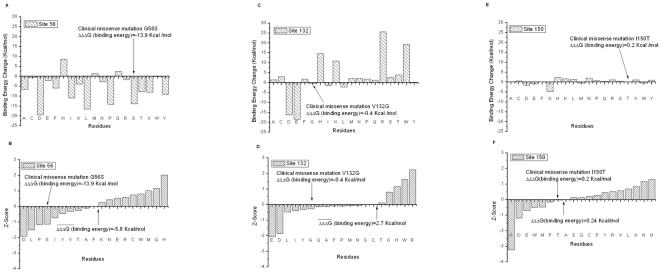
The results of structure-based energy calculations on the dimer affinity (binding free energy) with three different force field parameters (Charmm27, Amber98 and Oplsaa) and then results averaged for the clinically observed missense mutations G56S, V132G, and I150T, we used four force fields, which are Charmm27, Charmm19, Amber98 and Oplsaa, then results averaged). The left panels are the predicted energy changes of the binding free energy and the right panels are the Z-Scores of binding energy change. The 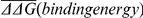 in the graph is the mean of binding free energy change over the 19 mutations. The results are shown for G56 (a–b), V312 (c–d) and I150 (e–f) sites.

Figure 3A shows the binding free energy changes per amino acid substitution. It can be seen that the vast majority of the mutations destabilize the dimer; however, at the same significant fraction of the rest of the substitutions has little effect. There is no clear trend with charge polarity, hydrophobicity, or the size of the side chain, which can be attributed to these two groups. In one case, a large hydrophobic group (Trp) causes almost no change in the binding affinity, while the Ala mutation is predicted to destabilize the dimer. A similar example is provided by the effects on substitution with Glu and Asp, both negatively charged, but predicted to cause a very different affect on the affinity. The analysis of the cases showed that the effect on the binding free energy strongly depends on the ability of the side chain to adopt a favorable geometry at the interface and equally important on the predicted effect on monomers stability (the effect of a mutation on the binding free energy is the difference between the effect on dimer and monomers stabilities).

The mean of the corresponding distribution (Figure 3B) is a large negative number (HSTD = 3.6 Kcal/mol) indicating that almost any substitution at G56 is predicted to decrease dimer affinity. Thus in terms of mutability, the site 56 is classified as a “non-tolerable” site. While there is no clear pattern to be able to determine which effect dominates (charge, hydrophobicity etc), the vast majority of the substitutions make the binding weaker. Therefore the site is classified as a “non-specific” site in terms of binding affinity, despite the prediction of the His substitution to increase affinity, since it is an isolated case. Although no clear pattern is observed, it is interesting to note that Asp is again the amino acid substitution with largest negative Z-score.

#### (b) V132 site

The V132 site is exactly at the dimer interface, but the side chain of the WT residue is parallel to it. In addition, the V132 side chain points toward a large cavity situated at the interface. As it was mentioned, the V132 position experiences a strong negative potential generated by neighboring acidic groups, which in the case of a dimer is stronger than the monomers, due to the contribution of the charges of the other monomer within the dimer.

In terms of the binding affinity, the site V132 is a classic example demonstrating the role of electrostatics on binding free energy. It can be seen (Figure 3C) that amino acid side chains carrying charge strongly affect the binding affinity, while no other mutation (except Trp) causes a change in the binding free energy. As indicated above, the position of V132 is at the bottom of the cleft formed between SMS monomers being in the dimer, where the pathway of the positively charged SPD coming into SMS is located. The strong negative potential at the V132 site is the reason why acidic group substitutions, Glu and Asp (Figure 3C), are predicted to greatly reduce dimer affinity. In contrast, positively charged groups are found to increase binding affinity. The effect of the Trp mutation is simply due to the hydrophobic effect, filling the cavity at the dimer interface with the bulky hydrophobic side chain of Trp.

The mean of the corresponding distribution (Figure 3D) is a positive number; however, it is mostly caused by several prominent contributions (positively charged side chains), while most of the remaining substitutions cause little effect on the binding affinity (HSTD  = 2.5 Kcal/mol). Because of that, the site V132 is classified as a “non-tolerable” but “specific” site. The effect strongly depends on the polarity of the charge of the side chain at position 132. Again, the largest negative Z-score is associated with Asp and Glu, while positively charged side chains are at the right side of the Z-score distribution.

#### (c) I150 site

The I150 site is far away from the interface and it is difficult to imagine any direct effect of the binding, including long range electrostatic interactions.

The predicted binding free energy changes are shown in Figure 3E. As anticipated, all substitutions have almost no effect on the binding affinity, with exception of the Gly mutant, which is obviously an artifact of our calculations. There is a slight tendency that negatively charged groups may destabilize the binding, while positively charged groups could make it tighter, but the effects are small, despite the long range of electrostatic interactions.

The mean of the corresponding distribution is almost zero indicating that this is a “tolerable” site (HSTD  = 0.8 Kcal/mol). However, due to the magnitude of the calculated changes, this site is classified as “specific” in terms of the binding affinity.

### Effects on pKa's and hydrogen bond network

**Figure 4:**
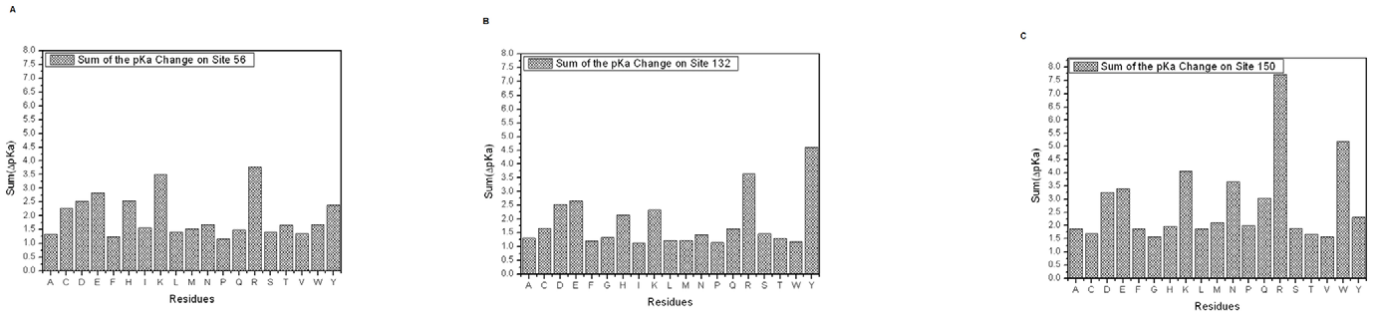
The pKa change due to each mutation for each monomer. The pKa shifts caused by each mutation for both C chain and D chain have been calculated using MCCE then results averaged.

Figure 4 shows the pKa changes induced by each amino acid substitution at each site. The vertical axis of the graph is the sum of the pKa change. This pKa change was calculated by the following formula:
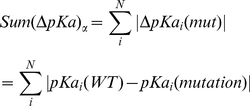
(7)where 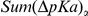 is the sum of the absolute value of the *i*th amino acid's pKa change due to the 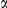
th mutation (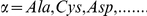) through all titratable residues (from 1 to N). In our case, N is equal to 139, which means that in each monomer there are 139 titratable amino acids. The results were averaged over C and D chains.

The calculated pKa shifts, which reflect reorganization of the hydrogen bond network upon each mutation, are shown in Figure 4. It can be seen that the hydrogen networks around sites 56 and 132 are predicted to be less sensitive to mutations, while substitutions at site 150 cause significant pKa shifts. This reflects the nature of the sites: the least sensitive is site 132 because it is less populated by titratable groups than other sites considered in this work. The site 56 has several titratable groups in the neighborhood, but they are relatively distant from the site. In addition, neither of these residues is involved either in catalysis or the binding, and thus does not directly contribute to the funding. In contrast, the site 150 is close to the active site region and a side chain at position 150 can establish new hydrogen bonds with residues participating in the hydrogen bond network involved in the catalytic reaction [16]. The largest is the effect of introducing titratable groups, because of the long range of electrostatics. The site 150 is situated in a negative potential environment and thus positively charged substitutions (Arg, Lys) are predicted to cause the largest pKa shifts. Specifically, the Arg, having a long side chain, can propagate toward the active site and affect the pKa and hydrogen bonding of active site residues and thus directly affecting the kinetics of the reaction.

With regard to the pKa calculations and following the classification scheme in the “Methods” section, site 56 is termed “non-sensitive specific”, site 132 is also “non-sensitive specific”, while site 150 is a “sensitive specific” site.

### In vitro experiments

Carrying experiments on all mutants investigated *in silico* would be too time-consuming. Instead we select 6 mutants from the *in silico* analysis including the clinically observed one. The selection of the mutants to be experimentally investigated is based on the following considerations: we pick up three groups mutations which are predicted to have strongest destabilizing/stabilizing effects on dimer affinity or monomer stability and a mutant that is predicted to have almost no effect (but to introduce an amino acid with drastically different physico-chemical characteristics from the wild type one). Such an approach let us explore the most extreme cases.

#### (a) G56 site

**Figure 5:**
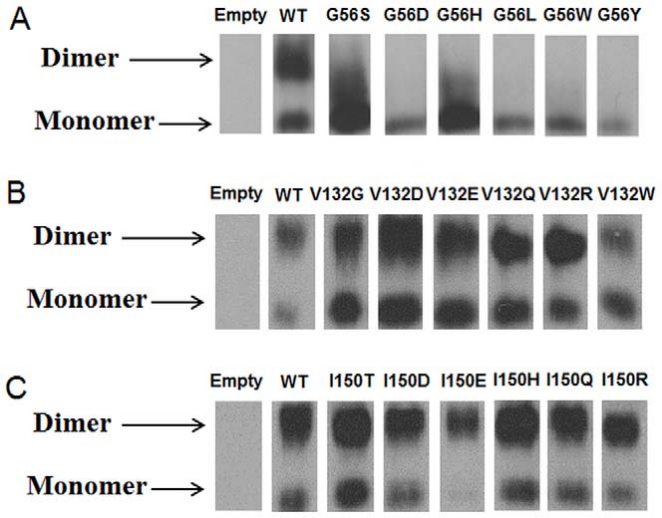
The experiments of gel electrophoresis for mutants at (a) G56 site; (b) V132site; (c) I150 site. The upper region represents the dimer and the lower region represents the monomer.

Mutations selected for *in vitro* experiments are: G56S (clinically observed mutation, predicted to have little effect of monomer stability, but to strongly destabilize the dimer); G56D (predicted to have no effect on monomer stability, but to lower drastically dimer affinity); G56H (predicted to stabilize bot the monomer and dimer interactions); G56L (expected to lower dimer affinity and have no effect on monomer stability); G56W (predicted to have no effect) and G56Y (expected to increase monomer stability but to lower dimer affinity). The in vitro experiments are shown in Figure 5A. Two distinctive patterns can be seen in Figure 5A: (1) cases indicating the presence of both monomers and dimers (WT and to certain extend, G56H) and (2) only monomer present (G56D, G56L, G56W and G56Y). In case of WT SMS, the dimer band is darker/larger than the monomer band (Figure 5A) indicating that the concentration of dimers is greater than of monomers. This confirms the previous experimental observation that dimer formation is crucial for the function of SMS (15). The clinically observed mutation, G56S, is predicted to lower dimer affinity and the effect is confirmed by *in vitro* experiments. Our *in silico* predictions for G56D, G56L and G56Y are also confirmed experimentally since no dimer band is present for these cases (Figure 5A). The G56D mutant deserves special attention since Asp56 is predicted by our pKa calculations to be protonated in the dimer, but to be ionized in the monomer, but our energy calculations were done assuming charged states for all titratable groups. The most closely related side chain to a protonated Asp in our analysis is Asn, which is predicted (but not so much as Asp) to lower dimmer affinity as well. Combined with the prediction that dimer formation will cause protonation of Asp56, which will require extra work, our calculations that Asp56 will destabilize the dimer are correct. The mutant G56H is interesting since it is the only one predicted to increase dimmer affinity and to increase monomer stability. The in vitro experiments (Figure 5A) show a broad band spanning from the monomer up to almost the dimer, but the “exact” dimer band is not populated. However, the analysis of the calculated pKa's reveals that His56 is not ionized in the dimer, while it is ionized in the monomer (pKa(monomer) = 7.1). This extra work to deprotonate His56 will reduce the predicted stabilizing effect on the dimer affinity. The experiments with the G56W mutant show no dimer formation, while our calculations predict no effect on both dimer affinity and monomer stability. This mutant was purposely selected for *in vitro* experiments because we were puzzled by the computational predictions that such a bulky side chain as Trp will cause no effect replacing Gly. Obviously, our computational protocol was incapable to account for all details for the experiment and made wrong prediction.

#### (b) V132 site

The mutants selected for in vitro experiments are: V132G (clinically observed mutation, predicted to destabilize the monomer by little and have no effect on dimer affinity); V132D, V132E (calculated to destabilize both the monomer and dimer stabilities); V132R and V132W (predicted to have little effect on monomer stability but to increase dimer affinity) and V132Q (predicted to have no effect). The clinically observed mutation, V132G, has similar pattern (Figure 5B) as the WT does. Both the monomer and dimer band are clearly seen in the gel experiments, which confirms our *in silico* predictions. All other mutants, except V132W, show no significant difference from the WT, i.e. both dimer and monomer bands are present, but the dimer band is more prominent. At first instance this can be viewed as failure of our *in silico* predictions, but analysis of the calculated pKa's reveals that Asp132, Glu 132 and Arg132 are neutral in the dimer (Table S4), while being fully ionized in the monomer. Since our predictions were made assuming fully ionized side chains of all titratable residues, but these residues are not ionized in the dimer. Therefore their effects should be similar to the effect caused by Gln132 mutant, which does not carry charge. This mutant is predicted *by in silico* analysis and confirmed by *in vitro* experiments not to affect dimer affinity (Figure 5B) as the neutral Asp132, Glu132 and Arg132 should be. The last mutant in our list is non titratable residue, V132W, and the experiments indicate that monomer population is larger than the dimer (in contrast to the WT). This is another case confirming our computational predictions, since V132W is predicted not to affect monomer stability, but not to decrease dimer affinity.

#### (a) I150 site

The list of selected mutants is: the clinically observed mutation I150T (which is predicted not to affect dimer affinity, but to decrease monomer stability), I150D, I150E, I150H, I150Q and I150R (all predicted not to have effect on dimer affinity, but to affect monomer stability). Figure 5C shows the results of *in vitro* experiments. It can be seen that none of the mutants, including clinically observed one, affects dimmer population. This confirms our predictions. However, the predicted effects on monomer stability cannot be directly evaluated from the experimental data. The presence of monomer band in other cases does not necessary indicate that the mutations do not have effect on monomer stability since the only thing that can be said is the dimer-monomer ratio. If the monomers are destabilized/stabilized, but the dimer affinity is unchanged, the ratio dimers/monomers will not change.

## Discussion

The investigation of the effect of missense mutations in SMS on the wild type properties of SMS was carried out with combined efforts of *in silico* modeling and *in vitro* experiments. Very good agreement between them was obtained for the cases being studied and the applicability of the selected experimental technique. At the same time, it was demonstrated also that correct predictions require taking into account the “correct” charge state of the ionizable groups. Especially in case of mutations that either are still not found in the general population or are very rare, the charged states of the mutated residues may be drastically different from their solutions values. This indicates the importance of pKa calculations and analysis of the charged states in the monomer and in the dimer.

The analysis showed that the mutability of three sites in SMS harboring missense mutations causing Snyder-Robinson Syndrome is quite different. The most “non-tolerable” is site I150, mutations at which are predicted by the folding free and pKa calculations to greatly disrupt WT properties of the SMS. Because of this, we predict that it is very unlikely that a nsSNP will be found at the I150 site. Instead, almost any mutation at site I150 is expected to cause SRS. On the other part of the spectrum is site V132. It is predicted by all means of our analysis to be “tolerable”. However, the calculated effects depend on the type of substitution. Thus, site V132 is capable of having either disease-causing or harmless mutations. The site G56 is in the middle with respect to its mutability. In terms of monomers stability and hydrogen bond effect, it is quite “tolerable”, but many mutations are predicted to affect dimer affinity. Since the dimer formation is essential for the function of the SMS, such mutations are expected to be disease-causing.

This investigation provides testable hypotheses which can be further tested against ever-growing databases of human nsSNPs, new clinical cases, or further *in vitro* experiments not reported in this work of stability/affinity of the SMS and NMR experiments on hydrogen bond networks in the SMS and mutants. It also indicates that missense mutations causing diseases do not mark the mutation site as disease-causing. Instead, the same mutation site may harbor harmless, nsSNPs, mutations.

## Supporting Information

Table S1 Results of folding free energy change calculations. **A** For site 56. Mean of the standard deviation over 19 mutations: 3.8 Kcal/mol; Half Standard (HSTD) = 1.9 Kcal/mol; **B** For site 132. Mean of the standard deviation over 19 mutations: 6.2 Kcal/mol; Half Standard (HSTD) = 3.1 Kcal/mol; **C** For site 150. Mean of the standard deviation over 19 mutations: 5.2 Kcal/mol; Half Standard (HSTD) = 2.6 Kcal/mol.
(DOCX)

Table S2 Results of the change of the binding free energy change calculations. **A** For site 56. Mean of the standard deviation over 19 mutations: 7.2 Kcal/mol; Half Standard (HSTD) = 3.6 Kcal/mol; **B** For site 132. Mean of the standard deviation over 19 mutations: 4.9 Kcal/mol; Half Standard (HSTD) = 2.5 Kcal/mol; **C** For site 150. Mean of the standard deviation over 19 mutations: 1.6 Kcal/mol; Half Standard (HSTD) = 0.8 Kcal/mol.
(DOCX)

Table S3 Site-directed mutation primers.
(DOCX)

Table S4 Results of pKa calculation for the two mutants. **A** pKa calculation for the dimer V132D (In the rebuilt pdb file, 3C6K, the corresponding residue number is V147D); **B** pKa calculation for the dimer V132E (In the rebuilt pdb file, 3C6K, the corresponding residue number is V147E).
(DOCX)
