# Supplementary material for: In Silico and In Vitro Investigations of the Mutability of Disease-Causing Missense Mutation Sites in Spermine Synthase
Source: PLoS One. 2011 May 27;6(5):e20373. doi: 10.1371/journal.pone.0020373 (PMC3103547; doi:10.1371/journal.pone.0020373)
Supplement: Table S1 — Results of folding free energy change calculations. A For site 56. Mean of the standard deviation over 19 mutations: 3.8 Kcal/mol; Half Standard (HSTD) = 1.9 Kcal/mol; B For site 132. Mean of the standard deviation over 19 mutations: 6.2 Kcal/mol; Half Standard (HSTD) = 3.1 Kcal/mol; C For site 150. Mean of the standard deviation over 19 mutations: 5.2 Kcal/mol; Half Standard (HSTD) = 2.6 Kcal/mol. (DOCX) [file pone.0020373.s001.docx]

**Table S1A**

| Mutation | Charmm27/19 | Amber98 | Oplsaa | Mean | Standard deviation |
| --- | --- | --- | --- | --- | --- |
| A | -0.9 | 1.0 | 6.6 | 2.2 | 3.9 |
| C | 0.4 | -2.4 | 8.1 | 2.0 | 5.4 |
| D | 2.1 | -2.4 | 1.8 | 0.5 | 2.5 |
| E | -0.4 | 1.5 | 8.2 | 3.1 | 4.5 |
| F | 2.9 | -0.0 | 0.4 | 1.1 | 1.6 |
| H | 5.4 | -0.1 | 3.5 | 3.9 | 2.8 |
| I | -2.7 | 3.4 | -0.4 | 0.1 | 3.1 |
| K | 10.4 | 3.1 | 17.1 | 10.2 | 7.0 |
| L | 0.2 | 0.0 | 0.9 | 0.4 | 0.5 |
| M | 3.3 | 2.5 | 9.4 | 5.1 | 3.8 |
| N | 3.2 | 3.1 | -3.1 | 1.1 | 3.6 |
| P | 0.6 | 4.3 | -4.7 | 0.1 | 4.6 |
| Q | -0.2 | -1.7 | 8.1 | 2.1 | 5.3 |
| R | 12.0 | 6.6 | 1.2 | 6.6 | 5.4 |
| S | 0.4/-7.9 | -0.5 | -3.1 | -2.8 | 1.8 |
| T | -1.0 | -2.6 | 2.9 | -0.2 | 2.8 |
| V | 0.9 | 3.2 | -8.0 | -1.3 | 5.9 |
| W | -4.5 | 6.2 | -4.1 | -0.8 | 6.1 |
| Y | 6.7 | 8.9 | 11.6 | 9.1 | 2.5 |

**Table S1B**

| Mutation | Charmm27/19 | Amber98 | Oplsaa | Mean | Standard deviation |
| --- | --- | --- | --- | --- | --- |
| A | 2.9 | -3.4 | 2.1 | 0.5 | 3.4 |
| C | 5.3 | -0.8 | 3.6 | 2.7 | 3.2 |
| D | -6.8 | -18.1 | 3.3 | -7.2 | 10.7 |
| E | -8.2 | -11.4 | 2.2 | -5.8 | 7.1 |
| F | -3.1 | -10.0 | 7.2 | -2.0 | 8.7 |
| G | 1.9/-11.6 | 1.6 | 4.8 | -1.1 | 2.4 |
| H | 14.0 | -4.5 | 12.0 | 7.2 | 10.2 |
| I | -3.8 | -7.7 | -0.5 | -4.0 | 3.6 |
| K | 3.0 | 0.6 | 12.2 | 5.2 | 6.1 |
| L | -1.8 | -14.6 | 6.7 | -3.2 | 10.7 |
| M | -2.1 | -2.7 | 10.5 | 1.9 | 7.4 |
| N | 2.0 | -5.0 | 4.3 | 0.4 | 4.8 |
| P | 5.6 | 3.3 | 4.8 | 4.6 | 1.2 |
| Q | -2.4 | -3.2 | 3.6 | -0.7 | 3.7 |
| R | -4.4 | -5.0 | 7.4 | -0.7 | 7.0 |
| S | 2.0 | 5.5 | -4.6 | 1.0 | 5.1 |
| T | 0.9 | -0.4 | 4.3 | 1.6 | 2.4 |
| W | -2.5 | -14.5 | 6.2 | -3.6 | 10.4 |
| Y | -3.2 | -11.1 | 8.8 | -1.8 | 10.0 |

**Table S1C**

| Mutation | Charmm19/27 | Amber98 | Oplsaa | Mean | Standard deviation |
| --- | --- | --- | --- | --- | --- |
| A | 5.9 | -10.6 | -1.7 | -2.1 | 8.2 |
| C | 2.7 | -6.0 | -1.2 | -1.5 | 4.3 |
| D | -24.3 | -17.9 | -20.2 | -20.8 | 3.3 |
| E | -17.8 | -9.0 | -17.3 | -14.7 | 5.0 |
| F | -6.6 | -5.4 | -4.3 | -5.4 | 1.1 |
| G | -2.9 | -13.4 | -4.9 | -7.1 | 5.6 |
| H | 7.7 | 13.1 | 4.5 | 8.4 | 4.4 |
| K | -1.0 | -1.7 | -14.5 | -5.7 | 7.6 |
| L | 0.4 | -5.0 | -6.5 | -3.7 | 3.6 |
| M | 1.3 | -4.6 | 0.8 | -0.9 | 3.3 |
| N | -1.1 | -14.9 | -1.2 | -5.7 | 7.9 |
| P | 1.2 | -2.8 | 0.6 | -0.3 | 2.2 |
| Q | 4.4 | -7.5 | 3.4 | 0.1 | 6.6 |
| R | 12.7 | 0.2 | -1.1 | 3.9 | 7.6 |
| S | -2.6 | -10.7 | -1.5 | -4.9 | 5.1 |
| T | -3.5/2.1 | -12.3 | -0.5 | -3.5 | 2.4 |
| V | -0.8 | -8.9 | 0.8 | -3.0 | 5.2 |
| W | 3.0 | -16.1 | 4.9 | -2.7 | 11.6 |
| Y | -9.6 | -7.2 | -12.9 | -9.9 | 2.8 |
