# Supplementary material for: In Silico and In Vitro Investigations of the Mutability of Disease-Causing Missense Mutation Sites in Spermine Synthase
Source: PLoS One. 2011 May 27;6(5):e20373. doi: 10.1371/journal.pone.0020373 (PMC3103547; doi:10.1371/journal.pone.0020373)
Supplement: Table S2 — Results of the change of the binding free energy change calculations. A For site 56. Mean of the standard deviation over 19 mutations: 7.2 Kcal/mol; Half Standard (HSTD) = 3.6 Kcal/mol; B For site 132. Mean of the standard deviation over 19 mutations: 4.9 Kcal/mol; Half Standard (HSTD) = 2.5 Kcal/mol; C For site 150. Mean of the standard deviation over 19 mutations: 1.6 Kcal/mol; Half Standard (HSTD) = 0.8 Kcal/mol. (DOCX) [file pone.0020373.s002.docx]

**Table S2A**

| Mutation | Charmm27/19 | Amber98 | Oplsaa | Mean | Standard deviation |
| --- | --- | --- | --- | --- | --- |
| A | -5.2 | -3.4 | -11.1 | -6.5 | 4.1 |
| C | -0.3 | -3.3 | 1.8 | -0.6 | 2.6 |
| D | -17.4 | -25.3 | -15.7 | -19.5 | 5.1 |
| E | -19.9 | -4.2 | 17.9 | -2.1 | 19.0 |
| F | -3.3 | -14.8 | 0.4 | -5.9 | 8.0 |
| H | -1.0 | 11.5 | 15.0 | 8.5 | 8.4 |
| I | -8.1 | -28.7 | 3.7 | -11.0 | 16.4 |
| K | -12.7 | -4.4 | 5.1 | -4.0 | 8.9 |
| L | -12.4 | -25.9 | -11.1 | -16.5 | 8.2 |
| M | -5.8 | 6.1 | 3.6 | 1.3 | 6.3 |
| N | -8.7 | -7.5 | 8.1 | -2.7 | 9.4 |
| P | -25.0 | -0.9 | -16.4 | -14.1 | 12.2 |
| Q | 0.7 | -2.8 | 9.5 | 2.4 | 6.3 |
| R | -8.2 | 2.5 | 0.8 | -1.6 | 5.8 |
| S | -17.0/-12.4 | -18.9 | -7.1 | -13.9 | 2.6 |
| T | -8.8 | -7.8 | -6.3 | -7.6 | 1.2 |
| V | -10.9 | -11.6 | -1.4 | -8.0 | 5.7 |
| W | 1.2 | -1.0 | -0.5 | -0.1 | 1.2 |
| Y | -7.2 | -16.4 | -3.7 | -9.1 | 6.6 |

**Table S2B**

| Mutation | Charmm27/19 | Amber98 | Oplsaa | Mean | Standard deviation |
| --- | --- | --- | --- | --- | --- |
| A | 2.1 | 0.4 | 1.7 | 1.4 | 0.9 |
| C | 4.3 | 1.0 | 3.5 | 2.9 | 1.7 |
| D | -14.9 | -16.6 | -17.4 | -16.3 | 1.3 |
| E | -10.7 | -21.1 | -23.6 | -18.5 | 6.9 |
| F | -5.8 | 8.0 | 3.2 | 1.8 | 7.0 |
| G | 4.3/-5.6 | -0.5 | -7.1 | -0.4 | 2 |
| H | -1.5 | 23.2 | 22.3 | 14.7 | 14.0 |
| I | -5.2 | 1.7 | -0.5 | -1.4 | 3.6 |
| K | 2.2 | 16.5 | 14.2 | 11.0 | 7.7 |
| L | -0.3 | -6.6 | 0.2 | -2.2 | 3.8 |
| M | 1.1 | 2.2 | 2.8 | 2.1 | 0.9 |
| N | -1.4 | 5.1 | 2.8 | 2.2 | 3.3 |
| P | -2.0 | 5.0 | 2.5 | 1.8 | 3.5 |
| Q | -1.2 | 1.2 | 3.9 | 1.3 | 2.5 |
| R | 9.9 | 36.0 | 31.0 | 25.7 | 13.9 |
| S | 3.5 | 0.2 | 4.0 | 2.6 | 2.0 |
| T | 3.4 | 3.4 | 5.0 | 3.9 | 0.9 |
| W | 22.3 | 10.0 | 25.5 | 19.3 | 8.2 |
| Y | -2.3 | -7.0 | 9.2 | -0.0 | 8.4 |

**Table S2C**

| Mutation | Charmm27/19 | Amber98 | Oplsaa | Mean | Standard deviation |
| --- | --- | --- | --- | --- | --- |
| A | 0.4 | -0.8 | 1.1 | 0.2 | 1.0 |
| C | 0.5 | 0.0 | 1.3 | 0.6 | 0.6 |
| D | -1.7 | -2.7 | -0.5 | -1.6 | 1.1 |
| E | -1.6 | -1.5 | 0.6 | -0.9 | 1.2 |
| F | -0.1 | -0.3 | 0.3 | -0.0 | 0.3 |
| G | -0.0 | 0.8 | -15.3 | -4.8 | 9.0 |
| H | 1.6 | 1.6 | 3.6 | 2.3 | 1.2 |
| K | 0.0 | 0.5 | 4.2 | 1.6 | 2.3 |
| L | 0.5 | 0.0 | 3.5 | 1.3 | 1.9 |
| M | -0.2 | -1.7 | 0.4 | -0.5 | 1.1 |
| N | -0.2 | 4.4 | 2.0 | 2.1 | 2.3 |
| P | -0.2 | 0.9 | 1.4 | 0.7 | 0.8 |
| Q | -0.1 | -0.3 | 1.7 | 0.4 | 1.1 |
| R | 0.4 | 1.6 | 1.2 | 1.0 | 0.6 |
| S | 0.2 | 0.2 | 1.0 | 0.4 | 0.5 |
| T | 0.3/-0.8 | -0.2 | 1.4 | 0.2 | 0.5 |
| V | 0.4 | 1.4 | 1.7 | 1.1 | 0.7 |
| W | -2.1 | -0.0 | 0.5 | -0.5 | 1.4 |
| Y | -0.5 | 0.1 | 3.2 | 0.9 | 2.0 |
