# Supplementary material for: In Silico and In Vitro Investigations of the Mutability of Disease-Causing Missense Mutation Sites in Spermine Synthase
Source: PLoS One. 2011 May 27;6(5):e20373. doi: 10.1371/journal.pone.0020373 (PMC3103547; doi:10.1371/journal.pone.0020373)
Supplement: Table S3 — Site-directed mutation primers. (DOCX) [file pone.0020373.s003.docx]

G56D FOR 5'-CCTACACAAACAAGAACGACAGCTTTGCCAATTTGAG-3'

G56D REV 5'-CTCAAATTGGCAAAGCTGTCGTTCTTGTTTGTGTAGG-3'

G56L FOR 5'-ATTTAGCAACCTACACAAACAAGAACCTAAGCTTTGCCAATTTGAGAATTTACCC-3'

G56L REV 5'-GGGTAAATTCTCAAATTGGCAAAGCTTAGGTTCTTGTTTGTGTAGGTTGCTAAAT-3'

G56W FOR 5'-GCAACCTACACAAACAAGAACTGGAGCTTTGCCAATTTGAGAATT-3'

G56W REV 5'-AATTCTCAAATTGGCAAAGCTCCAGTTCTTGTTTGTGTAGGTTGC-3'

G56H FOR 5'-GCAACCTACACAAACAAGAACCACAGCTTTGCCAATTTGAGAAT-3'

G56H REV 5'-ATTCTCAAATTGGCAAAGCTGTGGTTCTTGTTTGTGTAGGTTGC-3'

G56Y FOR 5'-TATTTAGCAACCTACACAAACAAGAACTATAGCTTTGCCAATTTGAGAATTTACCCA-3'

G56Y REV 5'-TGGGTAAATTCTCAAATTGGCAAAGCTATAGTTCTTGTTTGTGTAGGTTGCTAAATA-3'

V132D FOR 5'-CCGACGGGCGCCTGGATGAATATGACATAGAT-3'

V132D REV 5'-ATCTATGTCATATTCATCCAGGCGCCCGTCGG-3'

V132E FOR 5'-GCCGACGGGCGCCTGGAGGAATATGACATAGATGA-3'

V132E REV 5'-TCATCTATGTCATATTCCTCCAGGCGCCCGTCGGC-3'

V132Q FOR 5'-CCGCCGACGGGCGCCTGCAGGAATATGACATAGATGAA-3'

V132Q REV 5'-TTCATCTATGTCATATTCCTGCAGGCGCCCGTCGGCGG-3'

V132R FOR 5'-CGCCGACGGGCGCCTGCGTGAATATGACATAGATG-3'

V132R REV 5'-CATCTATGTCATATTCACGCAGGCGCCCGTCGGCG-3'

V132W FOR 5'-ACCGCCGACGGGCGCCTGTGGGAATATGACATAGATGAAG-3'

V132W REV 5'-CTTCATCTATGTCATATTCCCACAGGCGCCCGTCGGCGGT-3'

I150D FOR 5'-GTATATGACGAAGATTCACCTTATCAAAATGATAAAATTCTACACTCGAAGCAGTTTGGAAAT-3'

I150D REV 5'-ATTTCCAAACTGCTTCGAGTGTAGAATTTTATCATTTTGATAAGGTGAATCTTCGTCATATAC-3'

I150E FOR 5'-GTATATGACGAAGATTCACCTTATCAAAATGAGAAAATTCTACACTCGAAGCAGTTTGGAAAT-3'

I150E REV 5'-ATTTCCAAACTGCTTCGAGTGTAGAATTTTCTCATTTTGATAAGGTGAATCTTCGTCATATAC-3'

I150Q FOR 5'-GTATATGACGAAGATTCACCTTATCAAAATCAGAAAATTCTACACTCGAAGCAGTTTGGAAAT-3'

I150Q REV 5'-ATTTCCAAACTGCTTCGAGTGTAGAATTTTCTGATTTTGATAAGGTGAATCTTCGTCATATAC-3'

I150H FOR 5'-GTATATGACGAAGATTCACCTTATCAAAATCATAAAATTCTACACTCGAAGCAGTTTGGAAAT-3'

I150H REV 5'-ATTTCCAAACTGCTTCGAGTGTAGAATTTTATGATTTTGATAAGGTGAATCTTCGTCATATAC-3'

I150R FOR 5'-CGAAGATTCACCTTATCAAAATAGAAAAATTCTACACTCGAAGCAGT-3'

I150R REV 5'-ACTGCTTCGAGTGTAGAATTTTTCTATTTTGATAAGGTGAATCTTCG-3'
