# Supplementary material for: In Silico and In Vitro Investigations of the Mutability of Disease-Causing Missense Mutation Sites in Spermine Synthase
Source: PLoS One. 2011 May 27;6(5):e20373. doi: 10.1371/journal.pone.0020373 (PMC3103547; doi:10.1371/journal.pone.0020373)
Supplement: Table S4 — Results of pKa calculation for the two mutants. A pKa calculation for the dimer V132D (In the rebuilt pdb file, 3C6K, the corresponding residue number is V147D); B pKa calculation for the dimer V132E (In the rebuilt pdb file, 3C6K, the corresponding residue number is V147E). (DOCX) [file pone.0020373.s004.docx]

**Table S4A**

| pH | pKa/Em |
| --- | --- |
| NTR+C0001_ | 7.948 |
| HIS+C0005_ | 7.104 |
| HIS+C0006_ | 5.499 |
| HIS+C0007_ | 4.511 |
| HIS+C0008_ | 7.963 |
| HIS+C0009_ | 7.705 |
| HIS+C0010_ | 2.801 |
| ARG+C0017_ | 13.61 |
| ARG+C0020_ | 14 |
| HIS+C0021_ | 2.852 |
| ASP-C0025_ | 0 |
| LYS+C0031_ | 8.492 |
| ASP-C0033_ | 4.435 |
| GLU-C0035_ | 3.036 |
| LYS+C0039_ | 11.167 |
| GLU-C0047_ | 4.146 |
| GLU-C0052_ | 3.064 |
| HIS+C0055_ | 5.819 |
| ASP-C0059_ | 2.665 |
| HIS+C0060_ | 6.66 |
| TYR-C0062_ | 10.66 |
| TYR-C0066_ | 14 |
| LYS+C0069_ | 11.042 |
| ARG+C0077_ | 14 |
| TYR-C0079_ | 14 |
| HIS+C0081_ | 4.644 |
| ASP-C0087_ | 0 |
| TYR-C0091_ | 11.374 |
| ASP-C0092_ | 0 |
| ASP-C0094_ | 2.408 |
| LYS+C0098_ | 12.453 |
| GLU-C0099_ | 3.705 |
| GLU-C0100_ | 2.382 |
| ASP-C0102_ | 0.011 |
| LYS+C0107_ | 11.426 |
| GLU-C0109_ | 3.128 |
| GLU-C0110_ | 3.862 |
| ARG+C0111_ | 13.094 |
| LYS+C0113_ | 13.409 |
| GLU-C0114_ | 3.668 |
| ASP-C0118_ | 3.435 |
| ARG+C0122_ | 14 |
| LYS+C0124_ | 14 |
| ARG+C0125_ | 14 |
| ARG+C0131_ | 14 |
| ASP-C0136_ | 0 |
| ARG+C0137_ | 14 |
| TYR-C0138_ | 14 |
| ASP-C0143_ | 0 |
| ARG+C0145_ | 14 |
| ASP-C0147_ | 14 |
| GLU-C0148_ | 0 |
| TYR-C0149_ | 14 |
| ASP-C0150_ | 0 |
| ASP-C0152_ | 2.583 |
| GLU-C0153_ | 4.211 |
| TYR-C0156_ | 14 |
| ASP-C0157_ | 0.729 |
| GLU-C0158_ | 4.691 |
| ASP-C0159_ | 3.114 |
| TYR-C0162_ | 12.209 |
| LYS+C0166_ | 13.409 |
| HIS+C0169_ | 8.794 |
| LYS+C0171_ | 12.758 |
| ASP-C0182_ | 5.262 |
| GLU-C0187_ | 2.928 |
| ASP-C0189_ | 0 |
| TYR-C0192_ | 14 |
| ARG+C0194_ | 13.959 |
| LYS+C0201_ | 11.713 |
| GLU-C0202_ | 3.611 |
| ASP-C0203_ | 3.032 |
| TYR-C0204_ | 14 |
| LYS+C0207_ | 12.507 |
| ASP-C0208_ | 2.31 |
| ASP-C0216_ | 0 |
| CYS-C0221_ | 14 |
| GLU-C0222_ | 3.058 |
| LYS+C0225_ | 13.492 |
| LYS+C0227_ | 11.188 |
| LYS+C0229_ | 11.495 |
| GLU-C0235_ | 6.326 |
| ASP-C0237_ | 0.531 |
| ASP-C0242_ | 4.463 |
| CYS-C0244_ | 14 |
| LYS+C0245_ | 12.607 |
| LYS+C0246_ | 11.804 |
| TYR-C0247_ | 11.737 |
| ARG+C0249_ | 14 |
| LYS+C0250_ | 14 |
| CYS-C0252_ | 9.308 |
| ASP-C0254_ | 7.023 |
| ASP-C0257_ | 2.867 |
| LYS+C0260_ | 11.151 |
| ASP-C0262_ | 3.184 |
| CYS-C0263_ | 14 |
| TYR-C0264_ | 14 |
| GLU-C0269_ | 3.727 |
| ASP-C0270_ | 2.501 |
| CYS-C0271_ | 14 |
| LYS+C0276_ | 10.897 |
| ARG+C0277_ | 14 |
| TYR-C0278_ | 12.068 |
| LYS+C0280_ | 10.678 |
| GLU-C0281_ | 3.924 |
| ARG+C0283_ | 14 |
| GLU-C0284_ | 3.675 |
| ASP-C0286_ | 0.924 |
| TYR-C0287_ | 14 |
| ASP-C0291_ | 10.139 |
| GLU-C0302_ | 4.466 |
| GLU-C0303_ | 6.548 |
| ASP-C0304_ | 2.267 |
| GLU-C0308_ | 3.136 |
| ARG+C0311_ | 14 |
| ASP-C0315_ | 1.588 |
| LYS+C0319_ | 11.954 |
| LYS+C0322_ | 13.136 |
| ASP-C0324_ | 2.709 |
| LYS+C0326_ | 12.492 |
| TYR-C0327_ | 14 |
| CYS-C0333_ | 14 |
| GLU-C0338_ | 3.595 |
| TYR-C0343_ | 14 |
| GLU-C0344_ | 4.03 |
| GLU-C0345_ | 4.122 |
| ARG+C0349_ | 13.457 |
| TYR-C0351_ | 10.767 |
| CYS-C0352_ | 14 |
| GLU-C0355_ | 3.908 |
| LYS+C0358_ | 11.952 |
| GLU-C0359_ | 2.889 |
| TYR-C0366_ | 14 |
| GLU-C0368_ | 8.853 |
| TYR-C0373_ | 14 |
| LYS+C0377_ | 14 |
| LYS+C0378_ | 11.088 |
| LYS+C0380_ | 11.505 |
| CTR-C0381_ | 3.502 |
| NTR+D0001_ | 8.547 |
| HIS+D0005_ | 0 |
| HIS+D0006_ | 3.931 |
| HIS+D0007_ | 0 |
| HIS+D0008_ | 3.666 |
| HIS+D0009_ | 4.213 |
| HIS+D0010_ | 5.776 |
| ARG+D0017_ | 13.516 |
| ARG+D0020_ | 14 |
| HIS+D0021_ | 8.03 |
| ASP-D0025_ | 0 |
| LYS+D0031_ | 9.9 |
| ASP-D0033_ | 4.07 |
| GLU-D0035_ | 3.462 |
| LYS+D0039_ | 11.007 |
| GLU-D0047_ | 4.022 |
| GLU-D0052_ | 5.175 |
| HIS+D0055_ | 5.326 |
| ASP-D0059_ | 1.908 |
| HIS+D0060_ | 6.124 |
| TYR-D0062_ | 11.638 |
| TYR-D0066_ | 14 |
| LYS+D0069_ | 11.279 |
| ARG+D0077_ | 14 |
| TYR-D0079_ | 13.651 |
| HIS+D0081_ | 4.334 |
| ASP-D0087_ | 0 |
| TYR-D0091_ | 11.601 |
| ASP-D0092_ | 0 |
| ASP-D0094_ | 2.195 |
| LYS+D0098_ | 12.049 |
| GLU-D0099_ | 4.003 |
| GLU-D0100_ | 2.987 |
| ASP-D0102_ | 1.014 |
| LYS+D0107_ | 10.023 |
| GLU-D0109_ | 2.873 |
| GLU-D0110_ | 4.083 |
| ARG+D0111_ | 13.526 |
| LYS+D0113_ | 10.714 |
| GLU-D0114_ | 3.487 |
| ASP-D0118_ | 3.487 |
| ARG+D0122_ | 14 |
| LYS+D0124_ | 14 |
| ARG+D0125_ | 14 |
| ARG+D0131_ | 14 |
| ASP-D0136_ | 0 |
| ARG+D0137_ | 14 |
| TYR-D0138_ | 14 |
| ASP-D0143_ | 0 |
| ARG+D0145_ | 14 |
| ASP-D0147_ | 12.093 |
| GLU-D0148_ | 0 |
| TYR-D0149_ | 14 |
| ASP-D0150_ | 0 |
| ASP-D0152_ | 0.527 |
| GLU-D0153_ | 3.93 |
| TYR-D0156_ | 14 |
| ASP-D0157_ | 1.466 |
| GLU-D0158_ | 4.545 |
| ASP-D0159_ | 2.552 |
| TYR-D0162_ | 12.041 |
| LYS+D0166_ | 13.775 |
| HIS+D0169_ | 8.319 |
| LYS+D0171_ | 12.232 |
| ASP-D0182_ | 5.403 |
| GLU-D0187_ | 3.449 |
| ASP-D0189_ | 0 |
| TYR-D0192_ | 14 |
| ARG+D0194_ | 13.967 |
| LYS+D0201_ | 11.752 |
| GLU-D0202_ | 3.801 |
| ASP-D0203_ | 2.942 |
| TYR-D0204_ | 14 |
| LYS+D0207_ | 12.666 |
| ASP-D0208_ | 2.444 |
| ASP-D0216_ | 0 |
| CYS-D0221_ | 14 |
| GLU-D0222_ | 3.001 |
| LYS+D0225_ | 12.642 |
| LYS+D0227_ | 11.256 |
| LYS+D0229_ | 11.4 |
| GLU-D0235_ | 5.725 |
| ASP-D0237_ | 0 |
| ASP-D0242_ | 4.461 |
| CYS-D0244_ | 14 |
| LYS+D0245_ | 13.036 |
| LYS+D0246_ | 12.179 |
| TYR-D0247_ | 11.391 |
| ARG+D0249_ | 14 |
| LYS+D0250_ | 12.187 |
| CYS-D0252_ | 12.219 |
| ASP-D0254_ | 3.094 |
| ASP-D0257_ | 3.087 |
| LYS+D0260_ | 11.222 |
| ASP-D0262_ | 3.116 |
| CYS-D0263_ | 12.192 |
| TYR-D0264_ | 14 |
| GLU-D0269_ | 3.93 |
| ASP-D0270_ | 2.439 |
| CYS-D0271_ | 14 |
| LYS+D0276_ | 10.821 |
| ARG+D0277_ | 14 |
| TYR-D0278_ | 13.327 |
| LYS+D0280_ | 10.677 |
| GLU-D0281_ | 3.773 |
| ARG+D0283_ | 14 |
| GLU-D0284_ | 4.393 |
| ASP-D0286_ | 1.115 |
| TYR-D0287_ | 14 |
| ASP-D0291_ | 9.177 |
| GLU-D0302_ | 5.256 |
| GLU-D0303_ | 5.508 |
| ASP-D0304_ | 3.257 |
| GLU-D0308_ | 3.965 |
| ARG+D0311_ | 14 |
| ASP-D0315_ | 1.502 |
| LYS+D0319_ | 11.789 |
| LYS+D0322_ | 13.607 |
| ASP-D0324_ | 2.997 |
| LYS+D0326_ | 12.545 |
| TYR-D0327_ | 14 |
| CYS-D0333_ | 14 |
| GLU-D0338_ | 3.68 |
| TYR-D0343_ | 14 |
| GLU-D0344_ | 4.253 |
| GLU-D0345_ | 4.132 |
| ARG+D0349_ | 13.754 |
| TYR-D0351_ | 11.66 |
| CYS-D0352_ | 14 |
| GLU-D0355_ | 4.243 |
| LYS+D0358_ | 11.844 |
| GLU-D0359_ | 3.304 |
| TYR-D0366_ | 14 |
| GLU-D0368_ | 8.965 |
| TYR-D0373_ | 14 |
| LYS+D0377_ | 14 |
| LYS+D0378_ | 11.22 |
| LYS+D0380_ | 10.708 |
| CTR-D0381_ | 4.579 |

**Table S4B**

| pH | pKa/Em |
| --- | --- |
| NTR+C0001_ | 7.953 |
| HIS+C0005_ | 7.105 |
| HIS+C0006_ | 5.488 |
| HIS+C0007_ | 4.522 |
| HIS+C0008_ | 7.936 |
| HIS+C0009_ | 7.704 |
| HIS+C0010_ | 2.857 |
| ARG+C0017_ | 13.613 |
| ARG+C0020_ | 14 |
| HIS+C0021_ | 2.823 |
| ASP-C0025_ | 0 |
| LYS+C0031_ | 8.493 |
| ASP-C0033_ | 4.429 |
| GLU-C0035_ | 3.039 |
| LYS+C0039_ | 11.177 |
| GLU-C0047_ | 4.148 |
| GLU-C0052_ | 3.071 |
| HIS+C0055_ | 5.806 |
| ASP-C0059_ | 2.67 |
| HIS+C0060_ | 6.659 |
| TYR-C0062_ | 10.649 |
| TYR-C0066_ | 14 |
| LYS+C0069_ | 11.042 |
| ARG+C0077_ | 14 |
| TYR-C0079_ | 14 |
| HIS+C0081_ | 4.642 |
| ASP-C0087_ | 0 |
| TYR-C0091_ | 11.368 |
| ASP-C0092_ | 0 |
| ASP-C0094_ | 2.161 |
| LYS+C0098_ | 12.428 |
| GLU-C0099_ | 3.725 |
| GLU-C0100_ | 2.394 |
| ASP-C0102_ | 0 |
| LYS+C0107_ | 11.427 |
| GLU-C0109_ | 3.111 |
| GLU-C0110_ | 3.877 |
| ARG+C0111_ | 13.08 |
| LYS+C0113_ | 13.414 |
| GLU-C0114_ | 3.669 |
| ASP-C0118_ | 3.433 |
| ARG+C0122_ | 14 |
| LYS+C0124_ | 14 |
| ARG+C0125_ | 14 |
| ARG+C0131_ | 14 |
| ASP-C0136_ | 0 |
| ARG+C0137_ | 14 |
| TYR-C0138_ | 14 |
| ASP-C0143_ | 0 |
| ARG+C0145_ | 14 |
| GLU-C0147_ | 13.283 |
| GLU-C0148_ | 0.06 |
| TYR-C0149_ | 14 |
| ASP-C0150_ | 0 |
| ASP-C0152_ | 2.595 |
| GLU-C0153_ | 4.209 |
| TYR-C0156_ | 14 |
| ASP-C0157_ | 0.73 |
| GLU-C0158_ | 4.706 |
| ASP-C0159_ | 3.114 |
| TYR-C0162_ | 12.21 |
| LYS+C0166_ | 13.39 |
| HIS+C0169_ | 8.809 |
| LYS+C0171_ | 12.714 |
| ASP-C0182_ | 5.199 |
| GLU-C0187_ | 2.92 |
| ASP-C0189_ | 0 |
| TYR-C0192_ | 14 |
| ARG+C0194_ | 13.945 |
| LYS+C0201_ | 11.693 |
| GLU-C0202_ | 3.53 |
| ASP-C0203_ | 3.04 |
| TYR-C0204_ | 14 |
| LYS+C0207_ | 12.5 |
| ASP-C0208_ | 2.305 |
| ASP-C0216_ | 0 |
| CYS-C0221_ | 14 |
| GLU-C0222_ | 3.083 |
| LYS+C0225_ | 13.507 |
| LYS+C0227_ | 11.184 |
| LYS+C0229_ | 11.502 |
| GLU-C0235_ | 6.476 |
| ASP-C0237_ | 0.52 |
| ASP-C0242_ | 4.467 |
| CYS-C0244_ | 14 |
| LYS+C0245_ | 12.597 |
| LYS+C0246_ | 11.802 |
| TYR-C0247_ | 11.739 |
| ARG+C0249_ | 14 |
| LYS+C0250_ | 14 |
| CYS-C0252_ | 9.309 |
| ASP-C0254_ | 7.022 |
| ASP-C0257_ | 2.857 |
| LYS+C0260_ | 11.146 |
| ASP-C0262_ | 3.189 |
| CYS-C0263_ | 14 |
| TYR-C0264_ | 14 |
| GLU-C0269_ | 3.733 |
| ASP-C0270_ | 2.537 |
| CYS-C0271_ | 14 |
| LYS+C0276_ | 10.896 |
| ARG+C0277_ | 14 |
| TYR-C0278_ | 12.108 |
| LYS+C0280_ | 10.674 |
| GLU-C0281_ | 3.924 |
| ARG+C0283_ | 14 |
| GLU-C0284_ | 3.662 |
| ASP-C0286_ | 0.95 |
| TYR-C0287_ | 14 |
| ASP-C0291_ | 10.229 |
| GLU-C0302_ | 4.454 |
| GLU-C0303_ | 6.556 |
| ASP-C0304_ | 2.259 |
| GLU-C0308_ | 3.156 |
| ARG+C0311_ | 14 |
| ASP-C0315_ | 1.591 |
| LYS+C0319_ | 11.969 |
| LYS+C0322_ | 13.134 |
| ASP-C0324_ | 2.701 |
| LYS+C0326_ | 12.475 |
| TYR-C0327_ | 14 |
| CYS-C0333_ | 14 |
| GLU-C0338_ | 3.591 |
| TYR-C0343_ | 14 |
| GLU-C0344_ | 4.02 |
| GLU-C0345_ | 4.115 |
| ARG+C0349_ | 13.474 |
| TYR-C0351_ | 10.739 |
| CYS-C0352_ | 14 |
| GLU-C0355_ | 3.922 |
| LYS+C0358_ | 12.056 |
| GLU-C0359_ | 2.887 |
| TYR-C0366_ | 14 |
| GLU-C0368_ | 8.646 |
| TYR-C0373_ | 14 |
| LYS+C0377_ | 14 |
| LYS+C0378_ | 11.09 |
| LYS+C0380_ | 11.506 |
| CTR-C0381_ | 3.511 |
| NTR+D0001_ | 8.548 |
| HIS+D0005_ | 0 |
| HIS+D0006_ | 3.883 |
| HIS+D0007_ | 0 |
| HIS+D0008_ | 3.666 |
| HIS+D0009_ | 4.218 |
| HIS+D0010_ | 5.769 |
| ARG+D0017_ | 13.523 |
| ARG+D0020_ | 14 |
| HIS+D0021_ | 8.009 |
| ASP-D0025_ | 0 |
| LYS+D0031_ | 9.9 |
| ASP-D0033_ | 4.079 |
| GLU-D0035_ | 3.455 |
| LYS+D0039_ | 11.007 |
| GLU-D0047_ | 4.027 |
| GLU-D0052_ | 5.2 |
| HIS+D0055_ | 5.334 |
| ASP-D0059_ | 1.919 |
| HIS+D0060_ | 6.128 |
| TYR-D0062_ | 11.639 |
| TYR-D0066_ | 14 |
| LYS+D0069_ | 11.273 |
| ARG+D0077_ | 14 |
| TYR-D0079_ | 13.681 |
| HIS+D0081_ | 4.368 |
| ASP-D0087_ | 0 |
| TYR-D0091_ | 11.595 |
| ASP-D0092_ | 0 |
| ASP-D0094_ | 2.196 |
| LYS+D0098_ | 12.04 |
| GLU-D0099_ | 3.984 |
| GLU-D0100_ | 2.975 |
| ASP-D0102_ | 1.008 |
| LYS+D0107_ | 10.021 |
| GLU-D0109_ | 2.855 |
| GLU-D0110_ | 4.085 |
| ARG+D0111_ | 13.547 |
| LYS+D0113_ | 10.702 |
| GLU-D0114_ | 3.483 |
| ASP-D0118_ | 3.49 |
| ARG+D0122_ | 14 |
| LYS+D0124_ | 14 |
| ARG+D0125_ | 14 |
| ARG+D0131_ | 14 |
| ASP-D0136_ | 0 |
| ARG+D0137_ | 14 |
| TYR-D0138_ | 14 |
| ASP-D0143_ | 0 |
| ARG+D0145_ | 14 |
| GLU-D0147_ | 14 |
| GLU-D0148_ | 0 |
| TYR-D0149_ | 14 |
| ASP-D0150_ | 0 |
| ASP-D0152_ | 0.536 |
| GLU-D0153_ | 3.941 |
| TYR-D0156_ | 14 |
| ASP-D0157_ | 1.466 |
| GLU-D0158_ | 4.566 |
| ASP-D0159_ | 2.54 |
| TYR-D0162_ | 12.029 |
| LYS+D0166_ | 13.772 |
| HIS+D0169_ | 8.327 |
| LYS+D0171_ | 12.239 |
| ASP-D0182_ | 5.387 |
| GLU-D0187_ | 3.444 |
| ASP-D0189_ | 0 |
| TYR-D0192_ | 14 |
| ARG+D0194_ | 13.969 |
| LYS+D0201_ | 11.767 |
| GLU-D0202_ | 3.778 |
| ASP-D0203_ | 2.98 |
| TYR-D0204_ | 14 |
| LYS+D0207_ | 12.666 |
| ASP-D0208_ | 2.435 |
| ASP-D0216_ | 0 |
| CYS-D0221_ | 14 |
| GLU-D0222_ | 2.999 |
| LYS+D0225_ | 12.63 |
| LYS+D0227_ | 11.24 |
| LYS+D0229_ | 11.398 |
| GLU-D0235_ | 5.708 |
| ASP-D0237_ | 0 |
| ASP-D0242_ | 4.459 |
| CYS-D0244_ | 14 |
| LYS+D0245_ | 13.025 |
| LYS+D0246_ | 12.184 |
| TYR-D0247_ | 11.382 |
| ARG+D0249_ | 14 |
| LYS+D0250_ | 12.159 |
| CYS-D0252_ | 12.224 |
| ASP-D0254_ | 3.067 |
| ASP-D0257_ | 3.086 |
| LYS+D0260_ | 11.222 |
| ASP-D0262_ | 3.12 |
| CYS-D0263_ | 12.203 |
| TYR-D0264_ | 14 |
| GLU-D0269_ | 3.926 |
| ASP-D0270_ | 2.456 |
| CYS-D0271_ | 14 |
| LYS+D0276_ | 10.814 |
| ARG+D0277_ | 14 |
| TYR-D0278_ | 13.332 |
| LYS+D0280_ | 10.676 |
| GLU-D0281_ | 3.793 |
| ARG+D0283_ | 14 |
| GLU-D0284_ | 4.406 |
| ASP-D0286_ | 1.111 |
| TYR-D0287_ | 14 |
| ASP-D0291_ | 9.203 |
| GLU-D0302_ | 5.236 |
| GLU-D0303_ | 5.505 |
| ASP-D0304_ | 3.243 |
| GLU-D0308_ | 3.971 |
| ARG+D0311_ | 14 |
| ASP-D0315_ | 1.523 |
| LYS+D0319_ | 11.788 |
| LYS+D0322_ | 13.613 |
| ASP-D0324_ | 3.003 |
| LYS+D0326_ | 12.526 |
| TYR-D0327_ | 14 |
| CYS-D0333_ | 14 |
| GLU-D0338_ | 3.694 |
| TYR-D0343_ | 14 |
| GLU-D0344_ | 4.241 |
| GLU-D0345_ | 4.134 |
| ARG+D0349_ | 13.75 |
| TYR-D0351_ | 11.665 |
| CYS-D0352_ | 14 |
| GLU-D0355_ | 4.251 |
| LYS+D0358_ | 11.835 |
| GLU-D0359_ | 3.317 |
| TYR-D0366_ | 14 |
| GLU-D0368_ | 8.575 |
| TYR-D0373_ | 14 |
| LYS+D0377_ | 14 |
| LYS+D0378_ | 11.229 |
| LYS+D0380_ | 10.698 |
| CTR-D0381_ | 4.575 |
